# Correction: Direct chemical vapor deposition synthesis of large area single-layer brominated graphene

**DOI:** 10.1039/c9ra90038a

**Published:** 2019-05-22

**Authors:** Maria Hasan, Wang Meiou, Liu Yulian, Sami Ullah, Huy Q. Ta, Liang Zhao, Rafael G. Mendes, Zahida P. Malik, Nasir M. Ahmad, Zhongfan Liu, Mark H. Rümmeli

**Affiliations:** College of Chemistry & Molecular Engineering, Peking University Beijing 100871 P. R. China; College of Energy, Soochow Institute for Energy and Materials Innovations, Soochow University Suzhou 215006 China; Key Laboratory of Advanced Carbon Materials and Wearable Energy Technologies of Jiangsu Province, Soochow University Suzhou 215006 China; Institute of Environmental Technology, VŠB-Technical University of Ostrava 17. Listopadu 15 Ostrava 708 33 Czech Republic; IFW Dresden 20 Helmholtz Strasse Dresden 01069 Germany m.ruemmeli@ifw-dresden.de; Centre of Polymer and Carbon Materials, Polish Academy of Sciences M. Curie-Sklodowskiej 34 Zabrze 41-819 Poland; School of Natural Sciences, National University of Sciences and Technology Islamabad 44000 Pakistan; School of Chemical and Material Engineering, National University of Sciences and Technology Islamabad 44000 Pakistan

## Abstract

Correction for ‘Direct chemical vapor deposition synthesis of large area single-layer brominated graphene’ by Maria Hasan *et al.*, *RSC Adv.*, 2019, **9**, 13527–13532.

In the Acknowledgements section, the attribution “the Czech Republic from ERDF “Institute of Environmental Technology – Excellent Research” (No. CZ.02.1.01/0.0/0.0/15_019/0000853), should read “the Czech Republic from ERDF OP RDE project No. CZ.02.1.01/0.0/0.0/16_019/0000853”.

The Royal Society of Chemistry apologises for these errors and any consequent inconvenience to authors and readers.

## Supplementary Material

